# Barium zinc diarsenate

**DOI:** 10.1107/S1600536808025336

**Published:** 2008-08-09

**Authors:** Tamara Đordević

**Affiliations:** a Institut für Mineralogie und Kristallographie, Universität Wien-Geozentrum, Althanstrasse 14, A-1090 Vienna, Austria

## Abstract

The title compound, BaZnAs_2_O_7_, belongs to the family of isotypic series of compounds adopting the general formula *M*1^2+^ *M*2^2+^ *X*_2_O_7_ (*M*1^2+^ = Ca, Sr, Ba or Pb; *M*2^2+^ = Mg, Cr, Mn, Fe, Co, Ni, Cu, Zn or Cd; *X* = P or As). Suitable single crystals were prepared under hydro­thermal conditions. The framework structure is characterized by corner-sharing ZnO_5_ square pyramids and As_2_O_7_ groups where the Zn atoms occupy channels. X-ray diffraction analysis of single crystals twinned by non-merohedry [twin plane is (100)] yielded formula BaZnAs_2_O_7_. Raman spectra confirmed the presence of a non-linear As—O—As linkage.

## Related literature

For isostructural diarsenates, see: Pertlik (1986 ▶); Horng & Wang (1994 ▶); Wardojo & Hwu (1995 ▶); Chen & Wang (1996 ▶); Mihajlović *et al.* (2004 ▶). For the relationship to the known *M*1^2+^ *M*2^2+^ *X*_2_O_7_ compounds and the presence of non-merohedral twinning, see: Mihajlović *et al.* (2004 ▶). For related literature, see: Staack & Müller-Buschbaum (1998 ▶); Mihajlović & Effenberger (2006 ▶); Murashova *et al.* (1991 ▶); Nord *et al.* (1988 ▶).

## Experimental

### 

#### Crystal data

BaZnAs_2_O_7_
*M* *_r_* = 464.55
Monoclinic, 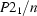

*a* = 5.6260 (10) Å
*b* = 8.557 (2) Å
*c* = 13.317 (3) Å
β = 90.01 (3)°
*V* = 641.1 (2) Å^3^
*Z* = 4
Mo *K*α radiation
μ = 20.08 mm^−1^
*T* = 293 (2) K
0.09 × 0.04 × 0.02 mm

#### Data collection

Nonius KappaCCD diffractometer
Absorption correction: multi-scan (Otwinowski & Minor, 1997 ▶) *T*_min_ = 0.265, *T*_max_ = 0.690
11068 measured reflections
2807 independent reflections
2747 reflections with *I* > 2σ(*I*)
*R*_int_ = 0.040

#### Refinement

*R*[*F* ^2^ > 2σ(*F* ^2^)] = 0.022
*wR*(*F* ^2^) = 0.056
*S* = 1.05
2807 reflections
102 parameters
Δρ_max_ = 2.21 e Å^−3^
Δρ_min_ = −1.77 e Å^−3^

### 

Data collection: *COLLECT* (Nonius, 2002 ▶); cell refinement: *SCALEPACK* (Otwinowski & Minor, 1997 ▶); data reduction: *DENZO-SMN* (Otwinowski & Minor, 1997 ▶; Otwinowski *et al.*, 2003 ▶); program(s) used to solve structure: *SIR97* (Altomare *et al*., 1997 ▶); program(s) used to refine structure: *SHELXL97* (Sheldrick, 2008 ▶) and *WinGX* (Farrugia, 1999 ▶); molecular graphics: *ATOMS* (Dowty, 2000 ▶); software used to prepare material for publication: *publCIF* (Westrip, 2008 ▶).

## Supplementary Material

Crystal structure: contains datablocks global, I. DOI: 10.1107/S1600536808025336/br2081sup1.cif

Structure factors: contains datablocks I. DOI: 10.1107/S1600536808025336/br2081Isup2.hkl

Additional supplementary materials:  crystallographic information; 3D view; checkCIF report

## Figures and Tables

**Fig. 1.**
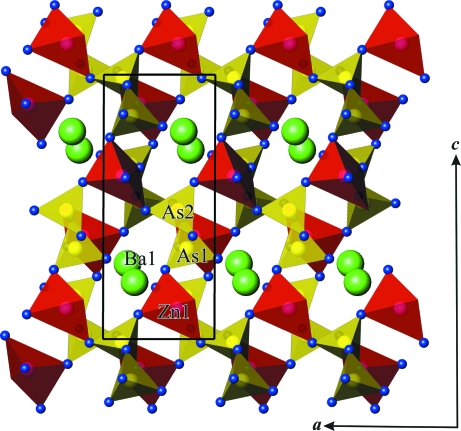
The crystal structure of BaZnAs2O7 in a projection parallel to [010].

**Fig. 2.**
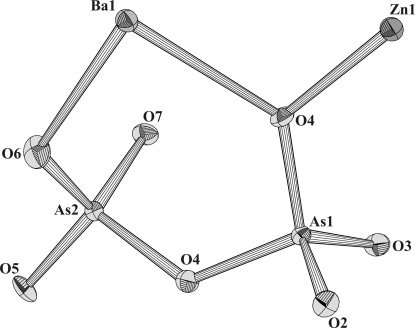
Atomic displacement ellipsoids at the 50% probability level.

## supplementary crystallographic information

### Comment

Diphosphates with the general formula M1M2P_2_O_7_ have been extensively studied during the last years. Among them are a couple of compounds with divalent M1 and M2 atoms (Mihajlović *et al.*, 2004 and references therein). These diphosphates show a variety of crystal structure types that are controlled by the distinct stereochemical behaviour of the M1 and M2 cations. It affects the coordination numbers, the degree of distortion of the coordination polyhedra, and the conformation of the P_2_O_7_ groups (Murashova *et al.*, 1991). By comparison much less is known about the structural chemistry of compounds with the formula M1^2+^M2^2+^*X*_2_O_7_ where *X* = V, Cr, Ge, As, and Si. Prior to this study, the crystal structures of only seven diarsenates M1^2+^M2^2+^As_2_O_7_ were known. Six of the first ones are isotypic with the title compound and crystallize in space group *P*2_1_/*n*: PbCuAs_2_O_7_ (Pertlik, 1986), SrCoAs_2_O_7_ (Horng & Wang, 1994), BaCuAs_2_O_7_ (Wardojo & Hwu, 1995; Chen & Wang, 1996), BaMgAs_2_O_7_, BaCoAs_2_O_7_ (Mihajlović *et al.*, 2004) and SrCuAs2O7 (Chen & Wang, 1996). Worthy to note is the five-coordinated *M*(2) position. CaCuAs2O7 has the same space-group symmetry; however, for the reduced cell the space group setting is *P*2_1_/*c* and the structural features are different (Chen & Wang, 1996; Staack & Müller-Buschbaum, 1998).

The crystal structure of BaZnAs_2_O_7_ is characterized by a three-dimensional framework formed from corner-sharing square pyramids ZnO_5_ and As_2_O_7_ groups. Within the framework bent chains running parallel to [010] are formed by the ZnO_5_ and As1O_4_ polyhedra. They are linked by the As2O_4_ tetrahedra. Each of the five corners of the square pyramids around the Zn atoms are shared with a different As_2_O_7_ group. The Ba position is located within channels parallel to [010] (Fig. 1). The Ba atoms are coordinated by nine oxygen atoms, and the coordination polyhedron can be described as a distorted tricapped trigonal prism. The average <Ba—O> bond length of 2.829 Å compare well to that of BaMgAs_2_O_7_, BaCoAs_2_O_7_ and BaCuAs_2_O_7_ (2.852, 2.829 and 2.852 Å, respectively). The pyroarsenate groups involve two crystallographically non-equivalent AsO_4_ tetrahedra. As expected, the longest As—O bonds are to the bridging oxygen atoms: <As1—O1> = 1.746 (2) Å and <As2—O1> = 1.751 (3) Å. The bond angles O_terminal_—As—O_terminal_ are significantly larger than O_bridging_—As—O_terminal_. The As1—O_bridging_—As2 angle is 124.67 (14)°.

In the 1000–800 cm^-1^ range, Raman spectrum shows the As—O antisymmetric and symmetric stretching modes of the (As_2_O_7_)^4-^ groups [904 (sh), 892 (*vs*), 847 (*m*), 825 (*m*)]. The bands at 784 (w) and 585 (*s*) cm^-1^ can be assigned to the asymmetric [ν_as_ (As—O—As)] and symmetric bridge stretching vibration [ν_s_ (As—O—As)], respectively and they are characteristic of the (As_2_O_7_)^4-^ group with a non-linear As—O—As bond (Nord *et al.*, 1988). In the region below 450 cm^-1^ appear the bending modes of the (As_2_O_7_)^4-^ groups, and various lattice modes of the compound.

The presence of only two structure types among M1^2+^M2^2+^-diarsenates contrasts with the situation among the M1^2+^M2^2+^-diphosphates where a greater variety of structure types is known; however, it probably reflects to some extent the different number of diphosphates and diarsenates studied so far.

### Experimental

Single crystals of BaZnAs_2_O_7_ were obtained as reaction products from mixtures of Ba(OH)_2_^.^8H_2_O (Merck, > 97%), 2ZnO^.^2CO_3_^.^4H_2_O (Alfa Products), and As_2_O_5_ (Alfa Products, > 99.9%). The mixture was transferred into Teflon vessel and filled to approximately 70% of their inner volume with distilled water (pH of the mixture was 2.5). Finally it was enclosed into stainless steel autoclave. The mixture was heated under autogeneous pressure from 293 to 493 K (4 h), held at that temperature (72 h) and rapidly cooled to room temperature. At the end of the reaction the pH of the solvent was 6. BaZnAs_2_O_7_ crystallizes as colourless, prismatic crystals up to 0.11 mm in length (yield *ca* 25%). It was obtained together with colourless, prismatic crystals of Ba(AsO_3_OH) (yield *ca* 60%) (Mihajlović & Effenberger, 2006) and uninvestigated amorphous mass yield *ca* 15%).

### Refinement

Several single crystals of the BaZnAs_2_O_7_ were studied with an automatic four-circle X-ray diffractometer equipped with a CCD area detector. Preliminary measurements showed sharp reflection spots and a pseudo-orthorhombic unit cell. However, closer inspections of the recorded CCD frames revealed that at higher diffraction angles some very slight splitting or slight broadening of the reflection spots was evident; this was later found to be due to twinning. The space-group symmetry was confirmed as *P*2_1_/*n* based on the extinction rules. It crystal structure was refined starting from the atomic coordinates given for BaCuAs_2_O_7_ (Chen & Wang, 1996). Although the structure models appeared to be crystal-chemically correct, the refinements initially converged unsatisfactorily. Distinct discrepancies between measured and calculated structure factors were observed (*F*_obs_^2^ > *F*_calc_^2^) for the most disagreeable reflections). The weighting scheme used by the programme *SHELXL97* (Sheldrick, 2008) suggested unexpectedly large values for the second weighting parameters. Furthermore, in the residual electron densities remained unusually high peaks which indicated the presence of 'phantom' atoms apparently mirroring atom positions across the (100) plane. Because of the pseudo-orthorhombic metrics of the unit cells (*β* close to 90°), non-merohedric twinning with a twin plane parallel to (100) was assumed. The application of the twin matrix (-1 0 0, 0 1 0, 0 0 1) during the refinement procedures reduced the *R*-values significantly. During the last stages anisotropic displacement parameters were allowed to vary for all atoms.

### Crystal data

**Table:**

BaZnAs_2_O_7_	*F*_000_ = 832
*M**_r_* = 464.55	*D*_x_ = 4.813 Mg m^−^^3^
Monoclinic, *P*2_1_/*n*	Mo *K*α radiation λ = 0.71073 Å
Hall symbol: -P 2yn	Cell parameters from 5551 reflections
*a* = 5.6260 (10) Å	θ = 0.4–35.0º
*b* = 8.557 (2) Å	µ = 20.08 mm^−^^1^
*c* = 13.317 (3) Å	*T* = 293 (2) K
β = 90.01 (3)º	Prismatic, colourless
*V* = 641.1 (2) Å^3^	0.09 × 0.04 × 0.02 mm
*Z* = 4	

### Data collection

**Table:**

Nonius KappaCCD diffractometer	2807 independent reflections
Radiation source: fine-focus sealed tube	2747 reflections with *I* > 2σ(*I*)
Monochromator: graphite	*R*_int_ = 0.040
*T* = 293(2) K	θ_max_ = 35.0º
φ and ω scans	θ_min_ = 1.5º
Absorption correction: multi-scan(Otwinowski & Minor, 1997)	*h* = −9→9
*T*_min_ = 0.265, *T*_max_ = 0.690	*k* = −13→13
11068 measured reflections	*l* = −21→21

### Refinement

**Table:**

Refinement on *F*^2^	Secondary atom site location: difference Fourier map
Least-squares matrix: full	*w* = 1/[σ^2^(*F*_o_^2^) + (0.0324*P*)^2^ + 1.09*P*] where *P* = (*F*_o_^2^ + 2*F*_c_^2^)/3
*R*[*F*^2^ > 2σ(*F*^2^)] = 0.022	(Δ/σ)_max_ = 0.001
*wR*(*F*^2^) = 0.056	Δρ_max_ = 2.21 e Å^−^^3^
*S* = 1.06	Δρ_min_ = −1.77 e Å^−^^3^
2807 reflections	Extinction correction: SHELXL97 (Sheldrick, 2008), Fc^*^=kFc[1+0.001xFc^2^λ^3^/sin(2θ)]^-1/4^
102 parameters	Extinction coefficient: 0.0037 (3)
Primary atom site location: structure-invariant direct methods	

### Special details

**Table:**

Geometry. All e.s.d.'s (except the e.s.d. in the dihedral angle between two l.s. planes) are estimated using the full covariance matrix. The cell e.s.d.'s are taken into account individually in the estimation of e.s.d.'s in distances, angles and torsion angles; correlations between e.s.d.'s in cell parameters are only used when they are defined by crystal symmetry. An approximate (isotropic) treatment of cell e.s.d.'s is used for estimating e.s.d.'s involving l.s. planes.

### Fractional atomic coordinates and isotropic or equivalent isotropic displacement parameters (Å^2^)

**Table:**

	*x*	*y*	*z*	*U*_iso_*/*U*_eq_	
Ba1	0.21674 (4)	0.34610 (2)	0.715139 (16)	0.01062 (5)	
Zn1	0.66892 (8)	0.14866 (4)	0.88747 (3)	0.01171 (8)	
As1	0.75049 (6)	0.03337 (3)	0.65727 (2)	0.00781 (6)	
As2	0.67177 (6)	0.31336 (4)	0.51369 (2)	0.00760 (6)	
O1	0.7114 (5)	0.1140 (3)	0.53795 (18)	0.0143 (4)	
O2	0.6667 (5)	−0.1533 (3)	0.6483 (2)	0.0130 (4)	
O3	1.0297 (5)	0.0613 (3)	0.6914 (2)	0.0175 (5)	
O4	0.5580 (4)	0.1208 (3)	0.73543 (19)	0.0111 (4)	
O5	0.8103 (5)	0.3354 (3)	0.40399 (19)	0.0117 (4)	
O6	0.3801 (5)	0.3486 (3)	0.5158 (2)	0.0114 (4)	
O7	0.8042 (5)	0.4073 (3)	0.60893 (18)	0.0131 (4)	

### Atomic displacement parameters (Å^2^)

**Table:**

	*U*^11^	*U*^22^	*U*^33^	*U*^12^	*U*^13^	*U*^23^
Ba1	0.01013 (8)	0.01003 (8)	0.01171 (8)	0.00076 (5)	−0.00048 (7)	−0.00098 (5)
Zn1	0.01165 (17)	0.01094 (16)	0.01254 (16)	−0.00053 (12)	−0.00077 (14)	0.00057 (11)
As1	0.00859 (13)	0.00678 (12)	0.00807 (12)	0.00043 (9)	−0.00037 (11)	0.00005 (9)
As2	0.00792 (12)	0.00819 (12)	0.00668 (11)	−0.00004 (10)	−0.00003 (10)	0.00028 (9)
O1	0.0249 (13)	0.0089 (9)	0.0092 (9)	0.0013 (9)	−0.0022 (9)	0.0006 (7)
O2	0.0166 (11)	0.0048 (9)	0.0176 (11)	−0.0001 (8)	0.0022 (9)	−0.0011 (7)
O3	0.0091 (10)	0.0163 (11)	0.0270 (13)	−0.0004 (8)	−0.0051 (9)	0.0005 (10)
O4	0.0105 (10)	0.0133 (10)	0.0097 (9)	0.0012 (8)	0.0021 (7)	−0.0029 (8)
O5	0.0094 (10)	0.0162 (10)	0.0096 (10)	0.0000 (8)	0.0035 (8)	0.0014 (7)
O6	0.0087 (10)	0.0145 (10)	0.0110 (10)	0.0005 (7)	0.0000 (8)	−0.0022 (8)
O7	0.0172 (10)	0.0110 (9)	0.0111 (9)	−0.0013 (9)	−0.0075 (8)	−0.0008 (7)

### Geometric parameters (Å, °)

**Table:**

Ba1—O3^i^	2.641 (3)	Zn1—O7^vi^	2.071 (3)
Ba1—O3^ii^	2.674 (3)	Zn1—O6^vii^	2.082 (3)
Ba1—O4	2.734 (3)	Zn1—O4	2.132 (3)
Ba1—O7^ii^	2.768 (3)	As1—O3	1.652 (3)
Ba1—O6	2.809 (3)	As1—O2	1.669 (2)
Ba1—O2^iii^	2.821 (3)	As1—O4	1.678 (2)
Ba1—O4^iii^	2.889 (3)	As1—O1	1.746 (2)
Ba1—O5^iv^	3.002 (3)	As2—O5	1.667 (3)
Ba1—O5^v^	3.157 (2)	As2—O6	1.669 (3)
Zn1—O2^i^	1.989 (2)	As2—O7	1.676 (2)
Zn1—O5^iv^	2.034 (3)	As2—O1	1.751 (2)
			
O3^i^—Ba1—O3^ii^	154.69 (6)	O5^iv^—Ba1—O5^v^	151.15 (6)
O3^i^—Ba1—O4	93.77 (8)	O2^i^—Zn1—O5^iv^	115.47 (11)
O3^ii^—Ba1—O4	69.23 (8)	O2^i^—Zn1—O7^vi^	145.21 (11)
O3^i^—Ba1—O7^ii^	124.18 (8)	O5^iv^—Zn1—O7^vi^	97.87 (11)
O3^ii^—Ba1—O7^ii^	77.40 (8)	O2^i^—Zn1—O6^vii^	85.52 (11)
O4—Ba1—O7^ii^	140.60 (7)	O5^iv^—Zn1—O6^vii^	118.44 (11)
O3^i^—Ba1—O6	105.27 (9)	O7^vi^—Zn1—O6^vii^	87.17 (10)
O3^ii^—Ba1—O6	91.37 (8)	O2^i^—Zn1—O4	90.21 (11)
O4—Ba1—O6	82.47 (7)	O5^iv^—Zn1—O4	79.64 (10)
O7^ii^—Ba1—O6	77.89 (8)	O7^vi^—Zn1—O4	86.10 (10)
O3^i^—Ba1—O2^iii^	96.19 (9)	O6^vii^—Zn1—O4	161.45 (10)
O3^ii^—Ba1—O2^iii^	77.12 (8)	O2^i^—Zn1—Ba1^viii^	142.43 (9)
O4—Ba1—O2^iii^	118.34 (7)	O5^iv^—Zn1—Ba1^viii^	59.85 (7)
O7^ii^—Ba1—O2^iii^	71.81 (8)	O7^vi^—Zn1—Ba1^viii^	48.98 (7)
O6—Ba1—O2^iii^	149.22 (8)	O6^vii^—Zn1—Ba1^viii^	130.97 (7)
O3^i^—Ba1—O4^iii^	67.32 (8)	O4—Zn1—Ba1^viii^	52.43 (7)
O3^ii^—Ba1—O4^iii^	123.92 (8)	O2^i^—Zn1—Ba1	77.81 (9)
O4—Ba1—O4^iii^	157.94 (3)	O5^iv^—Zn1—Ba1	51.31 (7)
O7^ii^—Ba1—O4^iii^	60.91 (7)	O7^vi^—Zn1—Ba1	120.23 (7)
O6—Ba1—O4^iii^	112.59 (7)	O6^vii^—Zn1—Ba1	150.26 (7)
O2^iii^—Ba1—O4^iii^	56.12 (7)	O4—Zn1—Ba1	44.28 (7)
O3^i^—Ba1—O5^iv^	82.60 (8)	O3—As1—O2	115.24 (14)
O3^ii^—Ba1—O5^iv^	72.34 (8)	O3—As1—O4	112.25 (14)
O4—Ba1—O5^iv^	55.22 (7)	O2—As1—O4	106.77 (13)
O7^ii^—Ba1—O5^iv^	132.28 (8)	O3—As1—O1	108.22 (15)
O6—Ba1—O5^iv^	137.57 (7)	O2—As1—O1	106.10 (13)
O2^iii^—Ba1—O5^iv^	66.11 (8)	O4—As1—O1	107.88 (13)
O4^iii^—Ba1—O5^iv^	108.88 (7)	O5—As2—O6	117.03 (14)
O3^i^—Ba1—O5^v^	70.18 (8)	O5—As2—O7	113.66 (13)
O3^ii^—Ba1—O5^v^	135.12 (8)	O6—As2—O7	109.72 (13)
O4—Ba1—O5^v^	133.79 (7)	O5—As2—O1	102.27 (12)
O7^ii^—Ba1—O5^v^	62.59 (7)	O6—As2—O1	107.34 (13)
O6—Ba1—O5^v^	62.25 (7)	O7—As2—O1	105.74 (12)
O2^iii^—Ba1—O5^v^	106.58 (7)	As1—O1—As2	124.67 (14)
O4^iii^—Ba1—O5^v^	52.15 (7)		

Symmetry codes: (i) −*x*+3/2, *y*+1/2, −*z*+3/2; (ii) *x*−1, *y*, *z*; (iii) −*x*+1/2, *y*+1/2, −*z*+3/2; (iv) *x*−1/2, −*y*+1/2, *z*+1/2; (v) −*x*+1, −*y*+1, −*z*+1; (vi) −*x*+3/2, *y*−1/2, −*z*+3/2; (vii) *x*+1/2, −*y*+1/2, *z*+1/2; (viii) −*x*+1/2, *y*−1/2, −*z*+3/2.
